# P-1528. *In Vitro* Activity of Omadacycline and Comparator Agents against a Collection of *Neisseria Gonorrhoeae* Urine Isolates Collected from the United States During 2018-2020

**DOI:** 10.1093/ofid/ofae631.1697

**Published:** 2025-01-29

**Authors:** S J Ryan Arends, J V Pierce, Holly Becker, Jill Lindley, Alisa W Serio, Mariana Castanheira

**Affiliations:** JMI Laboratories / Element, North Liberty, Iowa; Paratek Pharmaceuticals, King of Prussia, Pennsylvania; Element Materials Technology/Jones Microbiology Institute, NORTH LIBERTY, Iowa; Element Materials Technology/Jones Microbiology Institute, NORTH LIBERTY, Iowa; Paratek Pharmaceuticals, Inc., King of Prussia, Pennsylvania; JMI Laboratories, North Liberty, Iowa

## Abstract

**Background:**

Omadacycline (OMC) is a third-generation tetracycline (TET) class antibacterial approved for treatment of adults with acute bacterial skin and skin structure infections and community-acquired bacterial pneumonia caused by indicated organisms. OMC is active against bacterial isolates expressing common TET resistance mechanisms and strains resistant (R) to other drug classes. In this study, the *in vitro* activity of OMC and comparator agents was evaluated against a contemporary collection of *N. gonorrhoeae* clinical isolates. In addition, analysis of publicly available genomic sequences for these isolates was performed to determine the molecular mechanisms of TET resistance.

Antimicrobial activity of omadacycline and comparators tested against 100 Neisseria gonorrhoeae isolates

**Figure:**
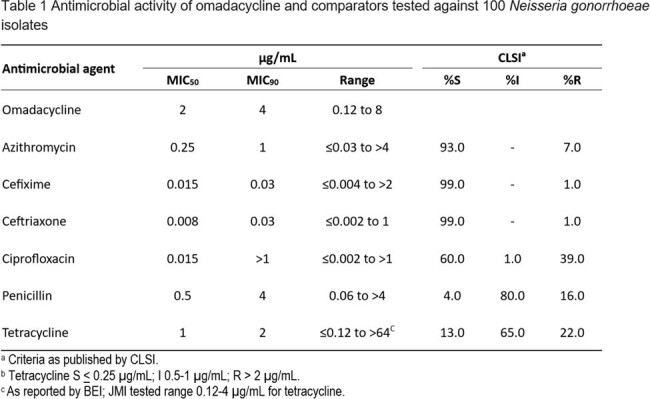

**Methods:**

The modal minimal inhibitory concentration (MIC) of OMC and comparator agents was determined by testing in triplicate by agar dilution for 100 *N. gonorrhoeae* clinical isolates (BEI Resources) collected from the urethra of patients with urethritis within the US (2018-2020). R or non-susceptible (NS) phenotypes were interpreted using breakpoints published by Clinical and Laboratory Standards Institute. Resistance genes were identified from genome sequences using ResFinder 4.0.

Cumulative distribution of omadacycline MIC values tested against comparator-resistant or non-susceptible N. gonorrhoeae subsets

**Figure:**
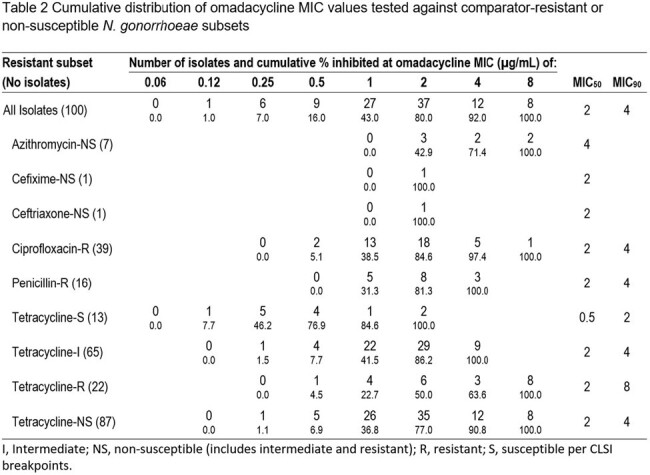

**Results:**

OMC displayed activity against *N. gonorrhoeae* isolates (MIC_50/90_, 2/4 µg/mL) that had limited susceptibility (S) to TET (13%), ciprofloxacin (CIP) (60%) and penicillin (PEN) (4%) but high S to azithromycin (AZM), cefixime (CFX), and ceftriaxone (CFN) (Table 1). Among isolates with NS or R phenotypes for comparators, the OMC MIC_50/90_ (when calculated) were generally not impacted, although some of these subsets had very low numbers (Table 2). The *rpsJ* mutation was widespread among TET-R isolates, including those with additional mutations in *porB*, the *mtrR* promoter and *tet(M)* (Table 3). The highest OMC MIC (8 µg/mL) correlated with combined mutations in *porB* and *rpsJ*. OMC had MICs < 1 µg/mL among isolates (n=3) with high level TET-R (MIC> 16 µg/mL), including those with *tet(M).*

Tetracycline resistance mechanisms among tetracycline resistant Neisseria gonorrhoeae isolates

**Figure:**
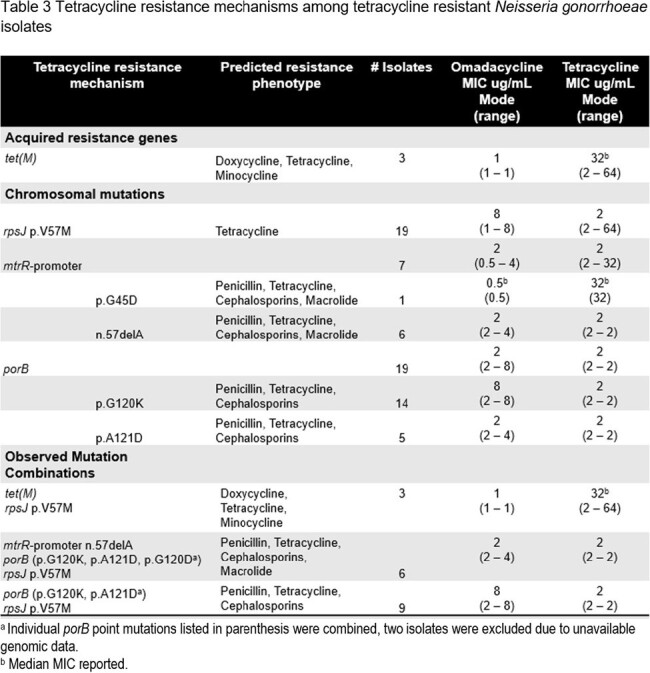

**Conclusion:**

OMC MICs were 0.12-8 µg/mL against this set of *N. gonorrhoeae* clinical isolates and the MIC distribution was minimally impacted by isolates NS/R to AZM, CFX, CFN, CIP, PEN, or TET. These results support the further research of OMC for *N. gonorrhoeae* infections, especially for those with high level TET-R.

**Disclosures:**

**J.V. Pierce, PhD**, Paratek Pharmaceuticals: Employee **Alisa W. Serio, PhD**, Paratek Pharmaceuticals: Employee

